# Asymmetrical nigral iron accumulation in Parkinson’s disease with motor asymmetry: an explorative, longitudinal and test-retest study

**DOI:** 10.18632/aging.103870

**Published:** 2020-09-27

**Authors:** Xiaojun Guan, Tao Guo, Cheng Zhou, Jingjing Wu, Ting Gao, Xueqin Bai, Hongjiang Wei, Yuyao Zhang, Min Xuan, Quanquan Gu, Peiyu Huang, Chunlei Liu, Baorong Zhang, Jiali Pu, Zhe Song, Yaping Yan, Feng Cui, Minming Zhang, Xiaojun Xu

**Affiliations:** 1 Department of Radiology, The Second Affiliated Hospital, Zhejiang University School of Medicine, Hangzhou, China; 2 Department of Neurology, The Second Affiliated Hospital, Zhejiang University School of Medicine, Hangzhou, China; 3 Department of Electrical Engineering and Computer Sciences, University of California, Berkeley, CA 94720, USA; 4 Institute for Medical Imaging Technology, School of Biomedical Engineering, Shanghai Jiao Tong University, Shanghai, China; 5 School of Information Science and Technology, ShanghaiTech University, Shanghai, China; 6 Helen Wills Neuroscience Institute, University of California, Berkeley, CA 94720, USA; 7 Department of Radiology, Hangzhou Hospital of Traditional Chinese Medicine, Hangzhou, China

**Keywords:** Parkinson’s disease, quantitative susceptibility mapping, iron, motor asymmetry

## Abstract

Parkinson’s disease (PD) is commonly characterized by asymmetrical motor impairment. This study aimed to clarify the iron distributions in PD patients with significant motor asymmetry and their longitudinal alterations. This study included 123 PD patients and 121 normal controls. Thirty-eight PD patients were revisited. PD patients with significant motor asymmetry were identified by using an objective criterion. Inter-group, inter-hemisphere and inter-visit differences of regional tissue susceptibility were analyzed. Iron accumulation in dominantly and non-dominantly affected substantia nigra (SN) were observed in PD patients with motor asymmetry compared with normal controls (p < 0.005, Bonferroni corrected). Iron accumulation in the dominantly affected SN was significantly higher than that in the non-dominantly affected SN (p < 0.01, Bonferroni corrected). After follow-up, time effect on the iron content in SN was observed, directing to decrease in PD patients with motor asymmetry without hemispherical difference (p < 0.05). In conclusion, asymmetrical iron accumulation in SN was associated with the motor asymmetry in PD at baseline, while along the disease evolution iron content in SN became longitudinally decreased. All these findings provide new evidence for PD pathogenesis that the abnormal iron metabolism in SN is complicated and not always unidirectional.

## INTRODUCTION

Parkinson’s disease (PD) is one of the most common neurodegenerative diseases; resting tremor, akinesia and rigidity are the typical and core symptoms in its clinical stage [1, 2]. Studies with large samples have shown that more than half of PD patients have an evident asymmetry of motor symptoms [3–5], and the asymmetry will persist along the disease trajectory [6, 7], which is important supportive evidence for PD diagnosis [2, 4]. However, the pathogenesis underlying such motor asymmetry in PD is still enigmatic. Therefore, the exploration of its underlying pathological basis would lead to a better understanding of PD since heterogeneous pathogenesis is becoming widely acknowledged in PD.

Limited but very significant postmortem evidence demonstrated a greater neuronal loss in substantia nigra (SN) contralateral to the initially dominantly affected limbs in PD [8], which is likely associated with the asymmetrical depletion of dopamine in striatum leading to the classic motor asymmetry in PD [9]. It has been well established that neuronal loss in the region of SN is always coupling with iron deposition [10, 11]. The excessive regional iron deposition is closely associated with the overload of oxidative stress [10, 12] and the aggregation of α-synuclein/Lewy body pathology [13, 14], both of which are the accepted cause of neuronal loss in PD. Therefore, the investigation of the asymmetry of regional iron distribution could be a promising alternative to reveal the underlying mystery of the motor asymmetry in PD.

In the last two decades, magnetic resonance imaging (MRI) studies have shed in vivo light on the possibility of constructing iron-related biomarkers for PD by measuring tissue relaxation properties or magnetic susceptibility [15–20]. However, whether the demonstrated asymmetrical neuronal loss is concordant with the asymmetrical iron deposition in the SN is still an open issue that needs to be well addressed. As historically documented, a couple of MRI studies reported significantly higher iron deposition in the SN contralateral to the dominantly affected limbs in comparison to that in the less affected SN [18, 20], while another study failed to reproduce this finding [19]. Several issues are possibly responsible for the inconsistencies. First and most importantly, no objective criteria were applied to identify the patients with exact asymmetrical symptoms. Second, the sample sizes included in these studies were relatively small. Last but not least, none of them tested the hypothesis in both PD patients with bilaterally asymmetrically affected limbs, and with unilaterally affected limbs (known as hemiparkinsonism [6, 21]).

In this study, in order to maximally control the sample bias, an objective criterion derived from a large PD population was applied to specifically identify PD patients that were showing significant motor asymmetry. We aimed to explore the iron distribution in the subcortical nuclei in those identified PD patients with motor asymmetry, and investigate the potential asymmetrical distribution of regional iron content. Additionally, a test-retest method was used to validate the robustness of the findings. Finally, a portion of PD patients were revisited and the longitudinal alterations of regional iron content were investigated.

## RESULTS

### Demographic and clinical information

**Figure 1:**
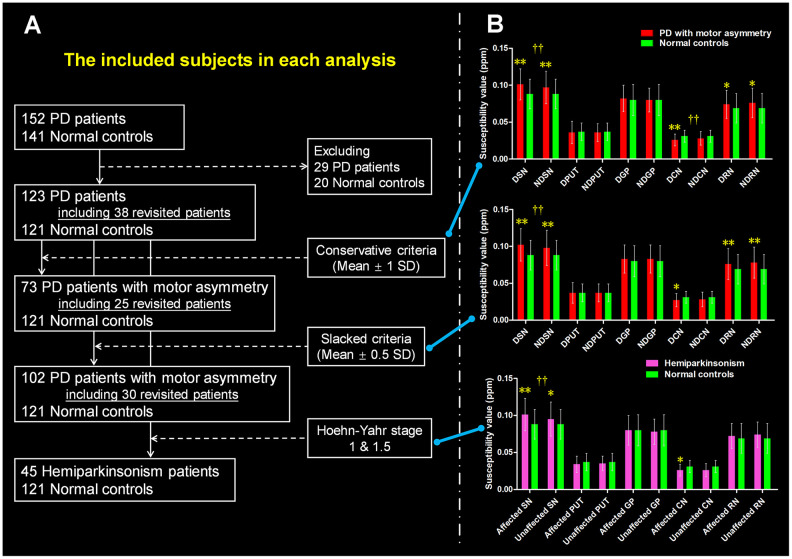
**The definition of PD patients with motor asymmetry and the intergroup difference of regional tissue susceptibility.** (**A**) The PD patients with significant motor asymmetry defined by using three objective criteria: conservative criterion, slacked criterion and hemiparkinsonism inclusion. (**B**) The inter-group differences between normal controls and PD patients with significant motor asymmetry that were defined by the three criteria mentioned above. */** are relating to the intergroup difference of regional tissue susceptibility. † is relating to the hemispherical difference (dominantly affected nucleus vs. non-dominantly affected nucleus) of regional tissue susceptibility. *: p < 0.05; **: p < 0.005 with Bonferroni correction. †: p < 0.05; ††: p < 0.01 with Bonferroni correction. PD = Parkinson’s disease; SN = Substantia nigra; PUT = Putamen; GP = Globus pallidus; CN = Caudate nucleus; RN = Red nucleus. DSN = Dominantly affected SN; NDSN = Non-dominantly affected SN; DPUT = Dominantly affected putamen; NDPUT = Non-dominantly affected putamen; DGP = Dominantly affected GP; NDGP = Non-dominantly affected GP; DCN = Dominantly affected CN; NDCN = Non-dominantly affected CN; DRN = Dominantly affected RN; NDRN = Non-dominantly affected RN; SD = Standard deviation.

The difference scores between the motor symptoms of left limbs and right limbs from the total 123 PD patients were normally distributed (mean ± 1 SD = -1.55 ± 7.71; p = 0.214). A summary of sample selection was shown in Figure 1A. For the conservative exploration (Table 1), 73/123 PD patients met the conservative criteria that patients with difference scores of ≥ 1 SD from the mean were defined as PD patients with significant motor asymmetry. Then, we observed that those PD patients were younger than normal controls (p = 0.021) while no significant difference in gender distribution was found between groups (p = 0.134).

**Table 1:** The demographic and clinical information for the participants.

**Variable**	**Normal controls**	**Parkinson’s disease**	**p_1_ value**	**Parkinson’s disease with motor asymmetry (mean ± 1 SD)**	**p_2_ value**	**Parkinson’s disease with motor asymmetry (mean ± 0.5 SD)**	**p_3_ value**	**Hemiparkinsonism**	**p_4_ value**
**Age**	60.97 ± 8.08	59.77 ± 8.29	0.253	58.20 ± 7.91	0.021	59.03 ± 8.10	0.076	56.39 ± 7.58	0.001
**Number, F/M**	121 (73/48)	123 (56/67)	0.021	73 (36/37)	0.134	102 (49/53)	0.066	45 (20/25)	0.067
**Symptom predominant, L/R**	-	-	-	31/42	-	46/56	-	16/29	-
**Disease duration,**	-	3.93 ± 3.78	-	3.63 ± 4.18	-	3.86 ± 3.99	-	2.87 ± 3.35	-
**Hoehn-Yahr stage**	-	1.87 ± 0.67	-	1.53 ± 0.57	-	1.78 ± 0.68	-	1.13 ± 0.22	-
**UPDRS III score**	-	25.19 ± 13.68	-	21.11 ± 12.42	-	23.27 ± 12.67	-	13.69 ± 6.03	-

All the data except the categorical variables (e.g., number and symptom predominant) are shown in mean ± SD.

P_1_ is relating to the intergroup comparison of the variables between normal controls and total Parkinson’s disease;

P_2_ is relating to the intergroup comparison of the variables between normal controls and Parkinson’s disease with motor asymmetry that were identified by using conservative criteria;

P_3_ is relating to the intergroup comparison of the variables between normal controls and Parkinson’s disease with motor asymmetry that were identified by using slacked criteria;

P_4_ is relating to the intergroup comparison of the variables between normal controls and hemiparkinsonism.

UPDRS = Unified Parkinson’s Disease Rating Scale; F/M = Female/Male; L/R = Left/Right; SD = Standard deviation; - = Not available.

Then, we made a slacker standard to include PD patients with a potential motor asymmetry. We included patients with difference scores of ≥ 0.5 SD from the mean, and finally, 103/123 PD patients met the criteria (Table 1). There was no significant intergroup difference in age or gender (p = 0.076 and p = 0.066, respectively).

A total of 45 PD patients were at the Hoehn-Yahr stages of 1 and 1.5 with unilateral limbs involved, termed hemiparkinsonism (Table 1). The age of these hemiparkinsonism patients was younger than that of the normal controls (p = 0.001). No significant difference in gender distribution was observed between them.

In the longitudinal investigations, we observed that, in both analyses, the disease stages (Hoehn-Yahr stages) were higher in PD patients with significant motor asymmetry at follow-up than at baseline, while the exact motor evaluations, Unified Parkinson’s Disease Rating Scale (UPDRS) III scores, were lower in those patients at follow-up than at baseline. Of note, PD patients at follow-up took a higher dose of Levodopa equivalent daily dose (LEDD) than they were at baseline. Details can be found in Table 2.

**Table 2:** Longitudinal demographic and clinical information for selected PD patients.

**Variable**	**PD Baseline**	**PD Follow-up**	**p_1_ value**	**PD with motor asymmetry (mean ± 1 SD)**			**PD with motor asymmetry (mean ± 0.5 SD)**		
				**PD Baseline**	**PD Follow-up**	**p_2_ value**	**PD Baseline**	**PD Follow-up**	**p_3_ value**
**Number, F/M**	38 (16/22)		-	25 (12/13)		-	30 (14/16)		-
**Time interval**	-	16.76 ± 5.68	-	-	17.52 ± 5.67	-	-	17.63 ± 6.08	-
**LEDD**	364.31 ± 311.48	564.47 ± 311.64	< 0.001	296.25 ± 244.83	502.21 ± 229.06	< 0.001	351.04 ± 317.58	537.26 ± 259.29	0.001
**Hoehn-Yahr stage**	1.72 ± 0.72	2.25 ± 0.55	< 0.001	1.34 ± 0.51	2.02 ± 0.42	< 0.001	1.55 ± 0.689	2.08 ± 0.44	< 0.001
**UPDRS III score**	24.18 ± 14.15	19.16 ± 13.40	0.006	19.00 ± 12.08	15.16 ± 9.22	0.078	21.20 ± 12.15	16.37 ± 9.70	0.016

All the data except the categorical variable (e.g., number) are shown in mean ± SD.

P_1_ is relating to the paired comparison of the variables between baseline and follow-up in the whole PD patients;

P_2_ is relating to the paired comparison of the variables between baseline and follow-up in the PD patients with motor asymmetry that were identified by using conservative criteria;

P_3_ is relating to the paired comparison of the variables between baseline and follow-up in the PD patients with motor asymmetry that were identified by using slacked criteria.

PD = Parkinson’s disease; F/M = Female/Male; LEDD = Levodopa equivalent daily dose; UPDRS = Unified Parkinson’s Disease Rating Scale; SD = Standard deviation.

### Conservative exploration: tissue susceptibility distribution in the subcortical nuclei

In the conservative exploration (Figure 1B), we observed significantly increased tissue susceptibility in both dominantly affected SN (p < 0.001) and non-dominantly affected SN (p = 0.003) in PD patients with significant motor asymmetry compared with normal controls, indicating iron overload in these regions. In addition, significantly decreased tissue susceptibility was observed in the dominantly affected caudate nucleus (CN) in PD patients with motor asymmetry compared with normal controls (p = 0.001). For the bilateral red nucleus (RN), tissue susceptibility had a trend towards increasing in PD patients in comparison with normal controls (p = 0.035 for dominantly affected side and p = 0.010 for the non-dominantly affected side respectively). No significant alteration of tissue susceptibility in other nuclei was observed in these PD patients.

Then, we observed that tissue susceptibility in the dominantly affected SN was significantly higher than that in the non-dominantly affected SN (t = 3.800, p < 0.001), while tissue susceptibility in the dominantly affected CN was lower than that in the non-dominantly affected CN (t = -2.703, p = 0.009) (Figure 1B). No significantly asymmetrical distribution of bilateral tissue susceptibility in the other nuclei was observed.

**Figure 2:**
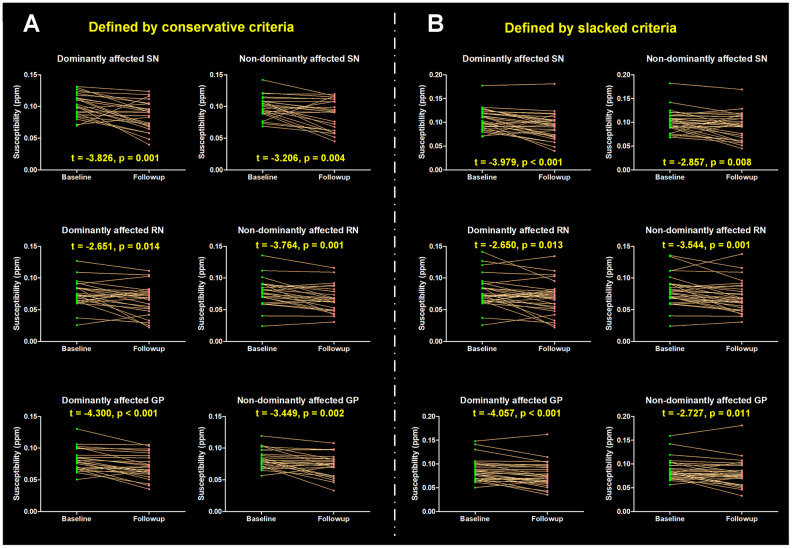
**Longitudinal alterations of regional tissue susceptibility showing statistical significance.** (**A**) PD patients with significant motor asymmetry were defined by conservative criteria according to the distribution of differences scores between bilateral motor scores (mean ± 1 SD), and 25 PD patients were included; (**B**) PD patients with significant motor asymmetry were defined by slacked criteria (mean ± 0.5 SD) and 30 PD patients were included. The post hoc t-test for each pair of regional tissue susceptibility at baseline and follow-up was conducted, t value and p value are shown. SN = Substantia nigra; GP = Globus pallidus; RN = Red nucleus.

This study also analyzed the interaction effect of time (a mean time interval of 17.52 months) and hemisphere (regional tissue susceptibility) in 25 revisited PD patients with significant motor asymmetry. As a result, the tissue susceptibility in iron-rich nuclei, like SN, RN and globus pallidus (GP), was found to be significantly decreased after follow-up (main time effect, all the p < 0.001), while no significant interaction effect was observed (p = 0.481, 0.927 and 0.862, respectively) in these regions, indicating similar iron alteration occurred along the disease evolution without the preference of affected hemispheres. No significant time effect or interaction effect on tissue susceptibility in the CN (p = 0.650 and 0.821) and putamen (p = 0.879 and 0.317) was observed. The post hoc analysis for each pair of regional tissue susceptibility at baseline and follow-up was shown in Figure 2A.

### Slacked exploration: tissue susceptibility distribution in the subcortical nuclei

In the slacked exploration (Figure 1B), PD patients had significantly higher tissue susceptibility in the bilateral SN than normal controls (both p < 0.001). Tissue susceptibility in the bilateral RN was significantly increased in PD patients compared with normal controls (p ≤ 0.001). Similar to the previous finding of iron alteration in CN, we observed decreased iron in the dominantly affected CN but did not reach the significance after multiple-comparison correction (p = 0.010 > 0.005). No significant alteration of tissue susceptibility in other nuclei was observed in PD.

In the PD patients with motor asymmetry, SN showed a significant and stable hemispheric asymmetry (Figure 1B). Specifically, the tissue susceptibility in the dominantly affected SN was significantly higher than that in its contralateral region (t = 3.923, p < 0.001). No significantly asymmetrical distribution of regional tissue susceptibility was found in the other nuclei.

Thirty PD patients were revisited with a mean time interval of 17.63 months. Similar to the findings in the conservative exploration, the tissue susceptibility in the SN, RN and GP was found to be significantly decreased after follow-up (main time effect, all the p < 0.001), while no significant interaction effect of the tissue susceptibility was observed (p = 0.397, 0.901 and 0.661, respectively) in these regions. No significant time effect and interaction effect on tissue susceptibility in the CN (p = 0.699 and 0.752) or putamen (p = 0.657 and 0.651) was observed. The post hoc analysis for each pair of regional tissue susceptibility at baseline and follow-up was shown in Figure 2B.

### Hemiparkinsonism: tissue susceptibility distribution in the subcortical nuclei

When comparing regional tissue susceptibility in the hemiparkinsonism with that in normal controls, a significantly increased tissue susceptibility was observed in the dominantly affected SN (p < 0.001) (Figure 1B). The statistical significance of the intergroup difference of tissue susceptibility in the non-dominantly affected SN and affected CN did not survive after Bonferroni multiple comparison correction (p = 0.022 and p = 0.010, respectively). Moreover, a significant asymmetrical distribution of tissue susceptibility in the SN was detected in these hemiparkinsonism patients (t = 3.775, p < 0.001). No significant asymmetrical distribution of bilateral tissue susceptibility was found in the other nuclei.

### Motor correlations of regional tissue susceptibility

No significant correlations of the regional tissue susceptibility showing significant inter-group differences and motor scores were observed.

## DISCUSSION

This study aimed to search the iron-related evidence to explain the motor asymmetry in PD patients that were defined by an objective criterion. Four main findings were reported: (1) In the explorations with conservative and slacked criteria, significant iron accumulation in both dominantly and non-dominantly affected SN was observed in PD patients with motor asymmetry compared with normal controls respectively; moreover, iron accumulation in the dominantly affected SN was significantly higher than that in the non-dominantly affected SN. (2) In a particular cohort of PD patients, hemiparkinsonism, with unilateral motor symptoms, significant iron accumulation was only found in the affected side of SN, and iron accumulation in this SN was significantly higher than that in its contralateral one. (3) Iron decline in the dominantly affected CN was observed in PD patients with motor asymmetry in an asymmetrical pattern, while iron accumulation in the bilateral RN was found but without asymmetrical distribution. (4) In the longitudinal investigations of PD patients with motor asymmetry, the main time effects on iron-rich nuclei (SN, RN, and GP) were observed, directing to decrease in PD patients with motor asymmetry, but, no interaction effects between time and hemisphere were investigated.

Motor asymmetry is a specific clinical characteristic for PD among the movement disorders, which is a supportive criterion for clinical diagnosis [2]. However, little is known about its underlying pathogenesis. Although motor asymmetry is highly acknowledged clinically, no objective or quantitative criterion for PD patients with motor asymmetry has been defined, making it challenging to clarify the underlying pathogenesis [18, 19]. This study provided an objective approach to identifying PD patients with significant motor asymmetry in a large PD population according to the distribution of difference scores between bilateral motor scores [22]. The application of this objective and quantitative criterion allows us to explore the regional iron alterations in the PD patients with defined motor asymmetry. Of note, both conservative and slacked criteria were used.

### Asymmetrical nigral iron accumulation in PD patients with significant motor asymmetry

First and the most important, we observed significant iron accumulation in both dominantly and non-dominantly affected SN in PD patients with motor asymmetry in comparison to normal controls, and iron accumulation in the dominantly affected SN was significantly higher than that in the non-dominantly affected SN when applying both conservative and slacked criteria. The excessive iron accumulation has been well related to accelerating the aggregation of α-synuclein/Lewy body pathology [13, 14], and exacerbating the iron-related oxidative damage in the SN [10, 12], which is the core hallmark of clinical PD [23]. Therefore, the asymmetrical iron accumulation between the dominantly affected and non-dominantly affected SN indicates that the pathological load is not evenly distributed in PD patients with significant motor asymmetry. Moreover, the significantly higher iron accumulation in the dominantly affected SN with potential higher pathological load may explain the greater neuronal loss in the SN contralateral to the dominantly affected limbs in PD [8]. Consistent to us, studies employing quantitative susceptibility mapping (QSM) and transcranial ultrasound reported asymmetrically increased tissue susceptibility and enlarged hyperechogenic area in SN of PD patients [18, 24]. However, He et al. did not find the asymmetrical distribution of nigral iron accumulation in PD using QSM [19]. We attributed such inconsistency to the lack of objective criterion to specifically identify PD patients with significant motor asymmetry, and to some extent, the small sample size also made the outcome bias.

It is worth noting that, through moving back to the early stage, on which PD patients merely suffer from unilateral motor impairment with one side of hemisphere possibly uninvolved (hemiparkinsonism), we also observed significant asymmetrical iron accumulation in the SN. Specifically, excessive iron accumulation was detected in the SN contralateral to the affected limbs, while the SN contralateral to the unaffected limbs was exempted from iron accumulation but with a trend. This finding further clues that the asymmetrical pathological load initiates early in PD, and the unilateral motor impairment may be accompanied with the iron accumulation in the contralateral SN.

Taken together, derived from the asymmetrical iron accumulation in SN in PD patients with significant motor asymmetry and hemiparkinsonism, we suggested that, from the initiation of neurodegeneration to the critical motor symptoms appearance, bilateral SN may not suffer from identical pathological loads, e.g., iron accumulation with its related oxidative damage or aggregation of α-synuclein/Lewy body pathology, that results in the asymmetrical neuronal loss leading to asymmetrical motor impairment.

### Iron alterations in CN and RN in the PD patients with motor asymmetry

Iron decline in the dominantly affected CN was observed in PD patients with motor asymmetry in an asymmetrical pattern. Very limited knowledge is available for the potential pathogenesis of iron alterations in CN, even though CN is one of the main dopaminergic innervations whose physiological function is disrupted secondary to the nigral degeneration [25]. In an independent legacy database that acquired seven years ago, we observed iron content in this region was negatively correlated with akinesia/rigidity severity in PD [26], which was in accordance with another study [27]. Thus, since akinesia/rigidity is the most common motor impairment in PD, iron decline in CN can be deduced from these negative clinical correlations, and its asymmetry further demonstrated the abnormal asymmetrical metabolism in this region.

Then we observed iron accumulation in bilateral RN but without significant asymmetrical distribution. Excessive iron accumulation in RN is not commonly related to the neuronal loss, but may be associated with the increased cerebellar compensatory capacity to the disrupted cerebral function [26, 28] in PD, because RN is known as a pivotal intersection between cerebellum and cerebrum [29, 30]. In agreement with our finding, a number of MRI studies have detected significant iron accumulation in this nucleus, and thus RN becomes the most frequently reported region following SN with remarkable iron overload in PD [17, 19, 26, 28, 31, 32]. The absence of asymmetrical accumulation of iron suggests the possibility that both of the bilateral cerebellar hemispheres participate in the functional compensation in PD patients with motor asymmetry.

In summary, the existence of iron decline in CN and iron accumulation in RN strongly demonstrated that the neurodegenerative process is not limited to the SN but involving multiple regions, showing heterogeneously abnormal iron metabolism in PD. For the purpose of deepening the understanding of the pathogenesis, future histopathological studies are needed to clarify the mechanism underlying the iron alterations in CN and RN in PD; of course, to what extent the sample heterogeneity will affect the effect size is needed to be especially answered.

### Longitudinal decline of iron in the iron-rich nuclei in PD

The evidence for longitudinal alterations of brain iron is still less elaborate. Currently, we observed that iron content in the iron-rich nuclei, e.g., SN, RN and GP, was dynamically decreased and no significant interaction effect between hemisphere and time was observed. Thus, an intriguing shift of regional iron distribution occurred in PD that iron accumulation at the baseline to iron decline along the disease progression, especially in SN and RN. Previous study cross-sectionally analyzed whole-brain iron distribution among PD patients with different disease severities and detected linear iron accumulation in SN, RN, and GP along the disease deterioration, providing preliminary evidence for the negative influence of regional iron accumulation on motor impairments [17]. In the present study, the revisited PD patients had significantly decreased UPDRS motor scores with receiving higher doses of anti-parkinsonian treatment, but their Hoehn-Yahr stages were dynamically increased, indicating that current anti-parkinsonian treatment don’t have disease modification effect [33], and PD pathology keeps progressing in the brain directing to involve bilateral limbs and impair the gait and postural stability [2]. Therefore, given the motor improvement after follow-up, iron purging in these nuclei (SN, RN and GP) might be expected; however, for the progressed disease stages, other pathologies otherwise brain iron that involve critical functional brain areas remain to be further clarified.

Dopamine and iron have been identified as a toxic couple and their coexistence would exacerbate the local oxidative stress in the SN [34]; thus, levodopa uptake not only relieves motor impairment, but also, to some extent, alleviates the load of dopamine production in the SN, resulting in the iron wash-out faster than pathological accumulation. Consistent with us, Du et al. reported that iron content in the SN was longitudinally reduced after a mean time interval of 18 months, and they suggested that the faster loss of iron is related to slower UPDRS III progression [35]; Wieler et al. observed that the significant iron accumulation in SN found at baseline was absent after 36 months’ follow-up [36], possibly indicating iron loss along the disease progression. However, the longitudinal results were not always invariable. Bergsland et al. and Ulla et al. investigated that progressive accumulation of iron in the SN occurred over three years in PD [37, 38]. For these inconsistencies, several possibilities might be responsible: (1) the disease heterogeneity that is widely acknowledged cannot be ignored, and small sample studies may have no ability to avoid this; (2) various anti-parkinsonian treatments are available for PD, which may have influence on the nigral iron accumulation as explained before. Taken together, these longitudinal findings give a better understanding of the dynamical iron-related nigral degeneration, providing new information about the complicated disease pathogenesis of PD. Thus future studies would benefit from the research design and result explanation.

This study had several limitations. First, because of no objective biomarker for diagnosing PD, pathological confirmation remains the golden standard. The PD diagnosis in the present study is made by a senior neurologist with more than 30 years’ clinical experience, and all included patients were assessed and diagnosed through longitudinal evaluations. Second, the sample size in the longitudinal analysis was relatively small, and possibly the results were influenced by various anti-parkinsonian treatments; thus, it is necessary to explore the roles of each kind of anti-parkinsonian drugs in modulating brain iron content in PD by future researches with larger sample sizes.

In conclusion, the asymmetrical iron accumulation in SN, indicating unidentical iron-related oxidative damage and aggregation of α-synuclein/Lewy body pathology in this region, was associated with the motor asymmetry in PD. Outside of SN, the existence of iron decline in CN and iron accumulation in RN demonstrated the heterogeneously abnormal iron metabolism in PD. And along the disease evolution, iron in the iron-rich nuclei (e.g., SN and RN) was found to be longitudinally decreased. All these findings provide new evidence for PD pathogenesis that the abnormal iron metabolism is complicated and not always unidirectional, but with temporal and spatial heterogeneity.

## MATERIALS AND METHODS

### Subject

All PD patients and normal controls, who were recruited from August 2014 to May 2018, signed informed consent forms in accordance with the approval of the Medical Ethics Committee of the Second Affiliated Hospital of Zhejiang University School of Medicine.

The diagnosis of PD was made by a senior neurologist (B. Z.) according to the United Kingdom PD Society Brain Bank criteria [4]. Participants with a history of neurologic or psychiatric disorders, brain trauma, or general exclusion criteria for MR scanning and analyzing were excluded. Basic demographic and clinical information, e.g., age, gender, disease duration and LEDD, and neurologic scales including UPDRS and Hoehn-Yahr stage were obtained from PD patients. For normal controls, basic demographic information was recorded.

Of note, 29 PD patients and 20 normal controls were further kicked out from the data analyses because of head motion, misregistration, significant brain atrophy, multiple microhemorrhage and clinical data absence. Finally, a total of 123 PD patients and 121 normal controls were included. Among these 123 PD patients, 38 patients were longitudinally revisited with a mean time interval of 16.76 months.

### Conservative definition of PD patients with significant motor asymmetry

Although a couple of previous studies had attempted to explore the asymmetrical distribution of nigral iron accumulation in PD [18, 19], no standardized definition protocol was mentioned, which might be linking to the inconsistent results. Here, we introduced a dichotomous approach to identify whether PD patients were showing significantly asymmetrical motor symptom [22]: (1) the total motor score from each affected limb (item 20 – item 26 of the UPDRS motor scale) was calculated; (2) the difference score between the bilateral total motor scores of each patient was obtained; (3) according to the distribution of difference scores, the patients with difference scores of ≥ 1 SD from the mean were identified as PD patients with significant motor asymmetry; (4) moreover, PD patients with hemiparkinsonism (Hoehn-Yahr stage = 1 and 1.5) but did not meet the criteria were still included; (5) finally, in those patients with a significantly asymmetrical motor symptom, we termed the subcortical nuclei contralateral to the dominantly affected limbs as dominantly affected nuclei. Therefore, a total of 73 PD patients (73/123) were found to have asymmetrical motor symptoms, and 25 patients (25/38) of them had longitudinal examinations.

### MRI scanning

All participants were scanned on a 3.0 Tesla MRI scanner (GE Discovery 750) equipped with an eight-channel head coil. During the scanning, the head was stabilized with restraining foam pads and earplugs were provided to reduce the noise. Enhanced susceptibility-weighted angiography (ESWAN) images were acquired using Gradient Recalled Echo sequence: repetition time = 33.7 ms; 1^st^ echo time/spacing /8^th^ echo time = 4.556 ms/3.648 ms/30.092 ms; flip angle = 20 degrees; field of view = 240 × 240 mm^2^; matrix = 416 × 384; slice thickness = 2 mm; slice gap = 0 mm; 64 continuous axial slices.

### QSM data processing

Susceptibility Tensor Imaging (STI) Suite V3.0 software package (https://people.eecs.berkeley.edu/~chunlei.liu/software.html) was used to calculate the susceptibility maps from the phase images as following: first, the raw phase was unwrapped using a Laplacian-based phase unwrapping and the normalized phase was calculated [39, 40]; then, the normalized background phase was removed using the spherical-mean-value filtering (V_SHARP) [41]; finally, tissue susceptibility was calculated using STAR-QSM (STreaking Artifact Reduction for QSM) method [42, 43]. The mean magnetic susceptibility of each individual brain was used as the susceptibility reference.

### Semi-automatic segmentation of subcortical nuclei

**Figure 3:**
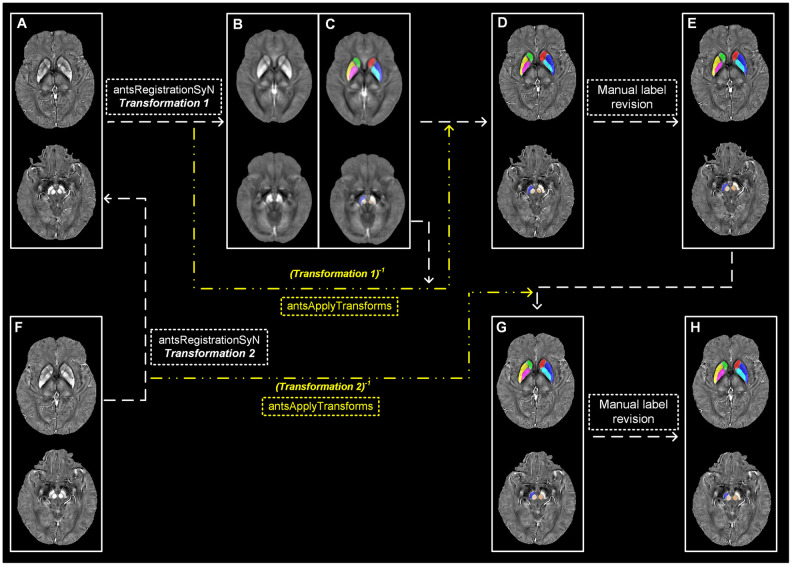
**Semi-automatic extraction of regional tissue susceptibility.** (**A**) Native QSM image at baseline; (**B**, **C**) QSM template ant the subcortical label in its space; (**D**) Warped subcortical label in the native QSM image at baseline; (**E**) Final label in the data analysis through manual revision; (**F**) Native QSM image at follow-up; (**G**) Warped subcortical label in the native QSM image at follow-up; (**H**) Final label in the longitudinal data analysis through manual revision. ANTs-SyN algorithms are used to complete the image coregistrations. QSM = Quantitative susceptibility mapping.

The tissue susceptibility of native subcortical nuclei in the basal ganglia and midbrain, including SN, RN, putamen, GP and CN, were extracted by using a semi-automatic segmentation method on the ANTs-R language environment (Figure 3A–3E): (1) by using ANTs-SyN coregistration algorithms [44], the native QSM image was registered to a newly constructed QSM template derived from a cohort of aging brains [45]; (2) the labels covering the subcortical nuclei were defined in the QSM template; (3) the labels in the QSM template were then warped to the native QSM image space by inverting the transformation matrix calculated in the first step; (4) manual refinement was performed to ensure the segmentation precision.

For the QSM images at follow-up, a semi-automatic registration-based segmentation was used as follows (Figure 3F–3H): (1) QSM image at follow-up was registered to that at baseline by using ANTs-SyN; (2) the subcortical labels obtained at baseline was then warped to the follow-up image by inverting the generated transformation matrix; (3) manual revision was conducted to ensure the segmentation precision.

### Statistical analysis

The one-sample Kolmogorov–Smirnov test was used to check the normality of the data. Differences in the age, UPDRS motor score, Hoehn-Yahr stage, disease course and gender distribution between groups were compared by using unpaired t-test, the Mann-Whitney U test and the Pearson chi-squared test appropriately. For the comparison of demographic and clinical information, e.g., LEDD, UPDRS III score and Hoehn-Yahr stage, between baseline and follow-up, paired t-test was used. Averaged regional tissue susceptibility from bilateral hemispheres in normal controls was calculated. General linear model was conducted to observe the intergroup difference of regional tissue susceptibility with age and gender as covariates. For these inter-group and inter-hemisphere comparisons of regional tissue susceptibility, Bonferroni multiple comparison correction was applied (p < 0.05/10 = 0.005 and p < 0.05/5 = 0.01, respectively). Subsequently, Repeated-measure ANOVA was used to evaluate the interaction effect between time and hemisphere. The post hoc t-test for each pair of regional tissue susceptibility at baseline and follow-up was conducted. Partial correlation analysis was conducted to detect the clinical relationship of the regional tissue susceptibility showing significant inter-group difference. P < 0.05 was regarded as statistically significant in general.

### Research extension and validation

### Extension to PD patients with hemiparkinsonism

As numerously documented, along the disease trajectory, most PD patients would experience a special stage (Hoehn-Yahr stage = 1 and 1.5), hemiparkinsonism [6, 21, 46], when motor symptoms only occur in the unilateral side of limbs while the other side of limbs is possibly unaffected. In this section, we aimed to extend previous findings to the patients in this special stage.

### Validation in an enlarged PD cohort by slacking the range of defined difference score

In consideration of the relatively low sample utilization (73/123) by using the conservative definition shown above (cut-off difference scores are ≥ 1 SD from the mean), we performed a pilot test by slacking the range of the cut-off difference scores into that of ≥ 0.5 SD from the mean, thus 102/123 PD patients were meeting the criteria. And among them, 30/38 patients were revisited. Here, we aimed to validate previous outcomes in a larger PD population.
